# Fail to prepare and you can prepare to fail: the experience of financing path changes in teaching hospitals in Iran

**DOI:** 10.1186/s12913-016-1405-7

**Published:** 2016-04-21

**Authors:** Leila Doshmangir, Arash Rashidian, Mehdi Jafari, Hamid Ravaghi, Amirhossein Takian

**Affiliations:** Iranian Center of Excellence in Health Management, School of Health Services Management, Tabriz University of Medical Sciences, Tabriz, Iran; Tabriz Health Management Research Center (NPMC), Tabriz University of Medical sciences, Tabriz, Iran; Department of Health Management and Economics, School of Public Health, Tehran University of Medical Sciences, Tehran, Iran; Knowledge Utilization Research Center, Tehran University of Medical Sciences, Tehran, Iran; School of Health Management and information Sciences, Iran University of Medical Sciences, Tehran, Iran; Health Management and Economics Sciences Research Center, Iran University of Medical Sciences, Tehran, Iran; Department of Global Health & Public Policy, School of Public Health, Tehran University of Medical Sciences, Tehran, Iran; College of Health & Life Sciences, Brunel University London, Uxbridge, UK

**Keywords:** Hospital autonomy, Purchaser-provider split, Healthcare reform, Implementation, Fee for service, Iran

## Abstract

**Background:**

In 1995, teaching and public hospitals that are affiliated with the ministry of health and medical education (MOHME) in Iran were granted financial self-sufficiency to practice contract-based relations with insurance organizations. The so-called “hospital autonomy” policy involved giving authority to the insurance organizations to purchase health services. The policy aimed at improving hospitals’ performance, hoping to reduce government’s costs. However, the policy was never implemented as intended. This was because most participating hospitals gave up to implement autonomous financing and took other financing pathways. This paper analyses the reasons for the gap between the intended policy and its execution. The lessons learned from this analysis can inform, we envisage, the implementation of similar initiatives in other settings.

**Methods:**

We conducted semi-structured interviews with 28 national and 13 regional health policy experts. We also gathered a comprehensive and purposeful set of related documents and analyzed their content. The qualitative data were analyzed by thematic inductive-deductive approach.

**Results:**

We found a number of prerequisites and requirements that were not prepared prior to the implementing hospital autonomy policy and categorized them into policy content (sources of funds for the policy), implementation context (organization of insurance organizations, medical tariffs, hospitals’ organization, feasibility of policy implementation, actors and stakeholders’ support), and implementation approach (implementation method, blanket approach to the implementation and timing of implementation). These characteristics resulted in unsuitable platform for policy implementation and eventually led to policy failure.

**Conclusions:**

Autonomy of teaching hospitals and their exclusive financing through insurance organizations did not achieve the desired goals of purchaser-provider split in Iran. Unless contextual preparations are in place, hospital autonomy will not succeed and problematic financial relations between service providers and patients in autonomous hospitals may not be ceased as a result.

## Background

Policymaking is an ongoing and dynamic process. Policies are usually subject to change, particularly in response to problems arising from their implementation [1]. Implementing a policy does not necessarily result in achieving the policy objectives [2]. Therefore, observing a gap between what policymakers intended and what they achieved is usual [3].

In the early 1990s, polices such as purchaser-provider split (PPS), internal competition, cost recovery and freedom of choice were introduced to focus on the demand (purchasing) side of the health systems [4, 5]. The main aim of such decentralizing policies was to reduce the role of the state; [6] divest itself of primary organizational responsibilities, and create independent sectors for service provision and financial responsibilities [7, 8].

Purchaser-provider split in healthcare was inspired by New Public Management approach to government [9, 10] based on principal-agent separation and contractual relationship between the purchaser (principal) and provider (agent) [11, 12]. Three levels of relationship are necessary for successful implementation of PPS: relationship with the public, with the provider and with the financiers who are responsible for distributing resources to the purchaser [9]. Failure in any of these links may overshadow the actual philosophy of PPS.

PPS may involve a “quasi” or “internal” market model, which seeks greater decentralization through creation of autonomous agencies to supply (provision) services [13, 14]. In the health system, separating ‘purchaser’ from ‘provider’ aims to increase the financial and managerial autonomy of hospitals and other providing organizations, and grant them greater authority to provide higher quality health care services [15]. This may lead to greater efficiency, extended users’ choice on the basis on needs-led services, and improved accountability of service providers [16–19].

There are a number of PPS experiences worldwide. In Sweden, PPS was primarily used to foster competition within the public sector and shift political control [20]. Australia [21], the National Health Service in the United Kingdom [22], New Zealand [23], and the United States of America are among countries that have implemented the PPS in their primary and hospital healthcare settings [18]. The first national attemmpt to implement PPS in primary health care in Iran during the implementation of family physicial program did not succeed in changing the status quo, became a reason for fighting, misunderstanding, lack of co-operation and failure of the fragile partnership between the purchaser and provider [24]. To date and after more than two decades of implementation in various settings, evidence shows that despite some modest benefits (improving public administration [25] and improving incentives for efficient production and service delivery [26], purchaser-provider split, has not sufficiently endured or fostered long term systematic changes [8] or greater efficiency in health care [27, 28].

### The Implementation of Purchaser-Provider split (PPS) in Iran

Until 1994, the main financing source for public hospitals (including teaching hospitals) in Iran was government-allocated public funds [29]. The ministry of health and medical education (MOHME) was the sole employer of healthcare providers, owned healthcare facilities, and purchased the services that were provided in public hospitals. Table 1 summarizes General information of Iranian health system in 1994 and 2013 Table 2.

**Table 1:** General information of Iranian health system (1994 and 2013)^a^ ^b^ ^c^(Iran Parliament research Center, 1994)

	1994	2013
Population	60,055,000	76, 921, 859
Total expenditure on health of GDP	7 %	5.6 %
Hospital bed index	1.5 beds per 1000 people	1.4 beds per 1000 people
Medical Insurance Coverage	40 %	83 %
Physician population	40266	137639

^a^Source: I.R. Iran,2013, http://www.amar.org.ir/default.aspx

^b^Source: I.R. Iran, MOHME, 2012. http://www.pooyasamaneh.net

^c^ http://www.behdasht.gov.ir/2013

**Table 2:** Typology of Iranian Hospitals according to their ownership^a^

Hospital affiliated to:	Hospitals	Hospital beds	Hospital beds (%)
MOHME	554	75569	67.8
Social security organization	70	9893	8.7
Armed forces and veterans	53	5005	4.4
Charities	30	3162	2.8
Other public hospitals	36	3034	2.7
Private	144	13076	11.5
Others	13	2424	2.1
Total	900	111363	100

^a^ Source: I.R. Iran, MOHME, 2012. http://www.pooyasamaneh.net

In November 1994, parliament approved the Medical Services Insurance (MSI (Act, implementing which was considered as a turning point for many significant changes within the Iranian health system. As advocated by the MSI Act, the Medical Services Insurance Organization (MSIO) was created in 1994 to provide universal health insurance for all citizens. With the aim of cutting financial relations between medical providers and patients in hospitals, the MSI Act’s mandate was splitting purchaser (MSIO) from provider (MOHME). The policy enforced the government to begin the implementation of universal medical insurance during the next five years times period. The MSIO was regulated by the Insurance High Council, one of whose duties was setting the medical tariffs as well as insurance premiums [30]. In 1995, the Parliament passed a bill enforcing public hospitals to fund themselves through revenue-generating fees (fee-for-services payment). Such a decision was also known as hospital autonomy policy [31]. During 1990–1993 and right before the time that the policy implementation started, average bed occupancy rate at public hospitals in Iran was about 56 %. The figure meant that over 16000 beds at the public hospital included in the study remained relatively unused [32]. Also the finding of performance assessment of 255 non-teaching hospitals (covering 30200 beds) that were affiliated with MOHME, brought a number of challenges with regard to performance and efficiency of such hospitals to the attention of policy makers [33].

The autonomous policy took away public hospitals’ funding from the MOHME’s public budget and replaced it with households’ co-payments as well as fee-for-service payment from the MSIO, so-called “Special Revenue Funds” [34]. The MSI Act stated that the MSIO must receive per capita premiums from annual governmental budget, and in return provide certain packages of hospital-based health services for citizens. By creating contractual-based relationships between the MSIO and public hospitals, the autonomy policy aimed to foster competition among hospitals; improve the quality of service delivery; increase efficiency, quality of health services and patients satisfaction [33], and as a result improve hospitals’ performance. Despite initial thoughts to implement the financial autonomy policy by approving MSI Act, the implementation of the policy did not meet the intention of its designers. To remedy the unexpected effects of policy implementation, parliament approved another policy in 1996, so-called the budget amendment plan of 30303. The new policy mandated the government to finance the salary of hospitals’ staff within medical universities annual budget, remunerated via public funds. This led public hospitals to enjoy three sources of funding:(1) the universities of medical sciences’ annual budget; (2) fee for services paid by medical insurance organizations; and (3) people’s out of pocket payment for services that were not covered by the MSIO. Plan 30303 ceased financial autonomy of hospitals.

This paper aims to answer two main questions:

1. Why the proposed hospital autonomy policy did not lead to purchaser-provider split in public hospitals in Iran?
2. What were the reasons for diversion of hospital autonomy policy from its intended routes?

In Iran, public health services are provided at three levels. At primary care level, Iran has a well-structured primary health care (PHC) network. Local health workers called Behvarzes are at the heart of basic healthcare services provision in rural Iran [35]. In the course of last decade, PHC has been strengthening through the implementation of family physician program in rural areas and cities of less than 20,000 populations [36]. Secondary and tertiary healthcare services in Iran are provided in hospitals, many of which are affiliated to the MOHME. The private sector mainly focuses on secondary and tertiary healthcare in urban areas [37]. There are 217 teaching hospitals in Iran. 68 % of beds in Iran’s public hospitals belong to 44 medical universities across the country, which are regulated by the MOHME.

Most health services in the Iranian public hospitals are purchased by various insurance organizations. These organizations provide universal or national health insurance, which is paid through a defined capitation premium, paid to them by the government.

The MSIO (currently called Health Insurance Organization: HIO) is considered the largest health insurance organization in Iran. In 2012, MSIO covered 36 million people (government employees, self-employed, rural inhabitants, etc.) [38]. Private insurance companies have a very small and negligible share in provision of basic health insurance in Iran. The private sector is conventionally prominent in providing complementary medical insurances in Iran.

## Methods

### Design and setting

This is a retrospective policy analysis using qualitative methods. We collected data (interviews and different types of documents) at national and regional levels of health system in Iran, from December 2012 until March 2013 and in two sequential phases.

### Sampling and data collection

We used purposeful and snowball sampling techniques to identify 42 interviewees. The interviewees were health system experts from across various capacities and positions: policy makers, hospital mangers and other key informants. The interviewees were included based on their accessibility, willingness to participate and provide information for this study, and diversity. Table 3 summarizes the characteristics of interviewees.

**Table 3:** The interviewees’ positions and distribution

Organization	Position	No
Ministry of health and Medical Organization(MOHME)	former Ministers of Health	4
	Advisor to minister	2
	Senior officers in medical tariff unit	2
	Senior policy officials in health policy making council	1
	Senior officials in Budget office	4
Iran’s Parliament	The Members of the 2^nd^, 3 ^rd^, 4^th^ Parliament	3
	Former senior national officials	2
	Former Senior policy officials in health Commission of Parliament	2
Insurance Organizations	Member of the Managing Board in Medical Services Insurance Organization	2
	Academic, Senior Policy Maker of the Iranian Health System	3
Hospitals	Hospital managers	3
	Head of financial affairs of hospitals	2
Universities of Medical sciences	Head of financial affairs of University	2
	University teachers	2
Vice-Presidency for Strategic Planning and Supervision (Former budgeting and Planning Organization)	Former Senior Policy officers	2
	Former Head of organization	1
Medical Council	Senior Policy officials	2
Health policy research centers	Health policy researchers and public policy researchers	3
Total		41

We used a reflexive interview guide (Appendix), which started from general questions and gradually moved to more specific questions. All interviews were undertaken at the interviewees’ working place. Interviews lasted between 48 and 142 min, were recorded and transcribed verbatim. In occasions that the interviewees paused because they were unable to remember particular policy events, we used exploratory questions to prompt responses.

In addition, we purposefully collected relevant documents of various types, from 1992 (before the approval of the MSI Act) to 1997 (one year after the approval of plan 30303) and analyzed their content. Table 4 shows the list of documents, gathered and analyzed in this study. Some documents were leading sources to other documents.

**Table 4:** List of documents, number and the sources of documents

Documents	Time period	Sources	No
Parliamentary proceedings	1991–1996	Parliament Libraries	43
Plans(fee-for-service Plan, 30303 Plan, hospitals modern System Plan (Novin Nezam in Farsi)	1992–1996	Parliament Libraries/Ministry of Health	3
the Five-Year Development Plans of Iran (the second, the Third, the Fourth and the Fifth)	1996–2000/2001–2006/2006–2011/2011- 2015	Parliament Libraries	4
the Iranian National Health Insurance proposal the Iranian National Health Insurance Act the Iranian National Health Insurance Bill	1989, 1992, 1994	Vice-Presidency for Strategic Planning and Supervision libraries	3
hospitals Administration regulations/bylaw	1979, 1994, 1995, 2007	MOHME	5
The budget Acts	(1989–1996), 2007	Islamic Consultative assembly research Center	8
Policy reports	1989–2010	Universities Libraries, Vice-Presidency for Strategic Planning and Supervision libraries, Parliament library	9
Policy articles/Text clippings	1991–2009	Vice-Presidency for Strategic Planning and Supervision libraries/Parliament library/local journals	29
Totals			104

### Data analysis

We used principles of content analysis (deductive-inductive) [39–41] as to analyze documentary data and merged this with data emerged from interviews. We started analysis by a preliminary thematic framework. Two authors (LD and AR) read all documents and transcripts several to extract relevant issues, themes and sub-themes. We continued this process until we developed a satisfactory thematic framework. The MAXQDA 10 software^1^ was used for the ease and more appropriate management of data set.

### Ethical considerations

Study objectives were clearly explained to all interviewees in advance and their oral consent was obtained to record interviews, and take notes from conversations should we stopped recording upon interviewees’ request. We ensured anonymity and observed data confidentiality with great care. The study participants provided consent to publish the participant identifiers. This research was approved by the Ethics Committee of the Iran University of Medical Sciences (Medical Ethics No: 92/n/105/2322).

## Results

We present our findings under three main themes: “policy content”, “implementation context”, and “implementation approach”. There are also nine subthemes and 33 issues, which are summarized in Table 5.

**Table 5:** The thematic framework explaining the themes, sub-themes and issues that represented the influential factors that influenced the development and implementation of hospital autonomy policy in Iran

Theme	Subthemes	Issues
Policy content	Sources of funds for the policy	-Capitation payment as employer contribution to government
		- Financial contribution of insured people
		- Government financial support
		- out of pocket health expenditure
Implementation context	Organization of insurance organizations	- An inexperienced insurance organization (MSIO)
		- Inadequate insurance fund
		- Inadequate health insurance coverage (population covered and depth of coverage)
		- delayed hospitals’ reimbursement
	Medical tariffs	-Technical aspects of setting medical tariffs
		- Delays in adjusting tariffs by general inflation
		- Stewardship and policy making in setting tariffs
	Organization of hospitals	- costs and revenues information system
		- teaching nature of target hospitals
		- imbalances in different aspects of autonomy (relative autonomy in generating revenues while no autonomy in staff management)
		- asymmetry of information between hospitals and insurance organizations
		- hospitals administration requirements
	Implementation feasibility	-lack of pilot study
		- no formal assessment of research evidence on autonomy implementation
		- no feasibility assessment
	Actors and stakeholders support	-lack of cooperation and coordination among various stakeholders
		- inadequate/ignorance of legal framework
		- cultural issues in hospitals
		- the interpretation of the policy
Implementation Approach	Implementation method	- top- down approach
		- expanding hospital autonomy before establishing its financing source (i.e., an effective universal health insurance organization)
	Blanket approach (‘one size fits all’ approach)	-Hospital Catchment Areas
		- Hospitals’ patients’ turnover
		-resources distribution among hospitals
	Timing of implementation	- difficult economic conditions (high national inflation rate)
		- hasty implementation
		- hospitals’ internal strategy for the implementation
		- optimistic time frames for policy success (expecting too much too soon)

Policy content refers to sources of funds for the policy development. “Implementation context“ refers to the contextual issues and planned steps for the policy development. “Implementation approach” refers to the issues that arose as a result of implementing the policy.

### The policy content

Hospital autonomy policy was implemented because of its perceived potential benefits to increase hospitals’ efficiency, and improve hospitals’ financial system [hospitals modern System Plan (Nezame Novin in Farsi)]. This intention of policy makers failed in reality. Meanwhile, despite the policy’s desire, the share of out of pocket (OOP) expenditures in financing of public hospitals went significantly up in Iran. We present the reasons for such unintended consequences below.

### Sources of funds for the policy implementation

Inadequate funding had severe adverse effects on the policy implementation. Two main sources of funds were available for hospitals’ autonomy: government’s contribution, paid as capitation payment to insurance companies, plus users’ co-payments. As stated in analyzed documents, many interviewees also pointed out that low capitation payments weakened the insurance market and hindered the implementation of the policy:

“*The Government was supposed to pay a lump sum from public funds to the newly established MSIO. However, the expenditure per capita was underestimated and did not correspond to the increasing costs of hospitals. This was a barrier to the implementation of fee-for-service policy*.” (Former minister of health).

Inadequate capitation payment, especially in the first years of the implementation of hospitals’ autonomy, pushed hospitals toward demanding greater copayments, which led to rapid increase of OOP. In other words, although public financial contribution should have been confined to premiums, “*autonomy policy forced hospitals to charge patients to compensate their monetary shortages”* [the Iranian National Health Insurance proposal], as illustrated by a senior officer from the former Budgeting and Planning Organization:

“*The Medical Services Insurance Act does not allow hospitals to receive money from people; it stipulates that people must pay insurance premiums, the government must finance insurance organizations, and insurance organizations must cover the costs of hospitals*.”

The policy highlighted the government’s contribution as a substantial proportion of funding for autonomous hospitals. However, this was never fully realized:

“*There was never sufficient financial support from the government (to run hospitals). Perhaps it would have been much easier to pursue the policy, if the government allocated a designated budget for implementing this particular policy*.” (National policy maker)

“*In 1995, 95 % of public hospitals’ income was from government’s contribution. The country’s current budget anticipates that 50 % of hospitals’ revenue must be generated from people co-payments and insurance organizations, which is not a realistic expectation*.” (Former minister of health)

### The implementation context

Our study demonstrated that inappropriate preparation was the leading cause for the pitfalls in the implementation of public hospitals’ autonomy policy in Iran. The implementation context, i.e., lack of infrastructures and other essential factors led to the PPS policy to fail. This is why the paper’s title is “you can prepare to fail”. Five subthemes were identified with regards to the implementation context: organization of insurance organizations, Medical tariffs, organization of hospitals, actors and stakeholders’ support, and the feasibility of implementation, as described below.

#### The organization of insurance organizations

Due to organizational weaknesses, the insurance industry could not generate sufficient revenue to fund autonomous hospitals. In addition, inadequate and delayed funds in the first years of MSIO establishment weakened this insurance organization:

*“The universal medical services insurance covered employees, rural residents, and self-employed and vulnerable individuals. To avoid any social tension, the policy allowed other people to get insurance with a low premium, once they admitted into a hospital! Although this was a valuable action to protect citizens, it was against principles and nature of insurance market, which is calculated on the basis of insurance premium and real medical tariffs. As a result, the government’s debt to insurance organizations increased, which adversely affected their financial ability*”. (Former senior policy officer).

Moreover, involved insurance organizations were not prepared enough for the implementation of the new policy, i.e., no proper mechanism was in place for hospitals’ reimbursements by insurances bodies:

“*The mere approval of the Universal health Insurance [MSI] Act did not lead to consistent insurance system, where the role of third party is fundamental. This policy is good in theory, but it is problematic in practice. What happened in reality was semi-autonomous administration of hospitals, as the government accepted to pay most expenses of hospitals*.” (Health policy researcher)

Despite the initial plans, universal medical services insurance was never implemented completely across the country. Instead, the implementation of hospital autonomy policy pushed public hospitals toward inappropriate revenue-generating methods, mainly OOP:

“*MSI Act was partially implemented and it did not cover all people, by implementing hospital autonomy policy, hospitals had to rely on out-of-pocket payments for their survival, and this was the main factor for increasing OOP payments*” (Advisor to the minister of health).

#### Medical tariffs

The MSI Act emphasized appropriate mechanisms to determine realistic tariffs for medical services, i.e., diagnostic and therapeutic services, taking into account the inflation rate and annual capitation. This did not happen in practice, which adversely affected day-to-day expenditure and total revenue of hospitals:

“*Wrong pricing system is one of the main reasons for unrealistic medical tariffs in Iran. The relative value of medical services is not accurately determined and most internal and external stakeholders are not accounted for calculating costs. There seemed to be a bias in pricing*.” (Senior medical tariff official).

The implementation of MSI Act policy undermined the authority of the MOHME in determining tariffs of medical services:

“*The ministry of health and Medical Education has few votes in the High Council of Medical Insurance. The MOHME ought to conduct strong lobbying to get money from insurance companies, and then distribute it among universities and hospitals*.” (Medical tariff’s officer).

#### Organization of target hospitals

The main context for implementing hospital autonomy policy was teaching hospitals. Many interviewees thought that the policy was inconsistent with the missions and goals of these types of hospitals. This was because such hospitals had to cover essential teaching and research costs, whereas the insurance organizations felt little responsibility to cover such expenditure.

“*The costs of rare and complex surgeries were extortionate for teaching hospitals. On the one hand, the hospitals must provide such services for teaching purposes. On the other hand, simpler and more profitable operations may be more desirable. We must admit that teaching hospitals were the wrong environment for the implementation of autonomy policy, which eventually lowered their scientific and educational merit*.” (Former minister of health).

Lack of proper accounting system in hospitals contributed to their delayed reimbursement by insurance organizations. In addition, information asymmetries between hospitals and insurance bodies led to problematic contracts for fee for service payments. Worse still, the public nature of hospitals reduced their competitiveness:

“*Prior to the implementation of MSI Act in 1995, the Ministry of Health’s income from hospitals was expected to be similar to the Ministry of Communication … It is wrong and illogical to compare communications with human lives*” (Former senior health policy maker).

In addition, the participating hospitals were granted incomplete and imbalanced autonomous power. The policy mostly focused on fiscal aspects of purchaser-provider split at the price of overlooking other crucial dimensions including Human resource capacity within target hospitals:

“*Most of our hospital managers were not educated in hospital management, health economics or other relevant disciplines. They are unable therefore to plan, manage costs, and oversee their organizations appropriately*” (Senior health officer).

#### Actors and Stakeholders’ support

Our findings endorsed the crucial role of various stakeholders, especially from the government, and their support in achieving the policy goals. Overall, heterogeneity and lack of harmony among involved stakeholders was the great concern for a number of interviewees:

“*The lack of coordination among stakeholders was phenomenal for implementing this (MSI Act) policy. The former Budgeting and Planning (currently: Vice-Presidency for Strategic Planning and Supervision libraries) Organization began to implement the policy without enough coordination with the Ministry of Health [and Medical Education]*.” (Health policy researcher).

The majority of interviewees pointed out the lack of cultural preparation and awareness as important factors that lowered stakeholders’ support for the policy. This was exacerbated by lack of legal support for the policy, which affected its desired implementation considerably:

“*If the entire country’s system, including the government and other state institutions abided by the MSI Act, many anticipated changes would have been definitely implemented during the set time period of the implementation*.” (Health policy maker).

#### Feasibility of the implementation

Many interviewees thought that conducting a feasibility analysis, e.g., looking for research evidence and conducing pilot studies to understand strengths and weaknesses of the policy, was a crucial step that was neglected prior to the implementation of hospitals’ autonomy policy. In other words, the main criticism was not taking similar experiences into account, as well as lack of engagement with health experts and practitioners to design the policy. A former senior health official commented:

“*They [former Budgeting and planning organization] should have ensured whether all prerequisites for implementing the policy were in place or not, and whether or not the policy could be completely implemented nationwide? Prior to its universal implementation, strengths and weaknesses, as well as opportunities and threats of the policy implementation must have been analyzed*.”

### The implementation approach

Here, we describe our participants’ views towards the implementation of hospital autonomy policy with regards to: method, approach, and timing of the implementation.

#### Method of the implementation

Some interviewees stated that top-down implementation of hospital autonomy, i.e., by direct command of the former Planning and Budgeting Organization, and without engaging with street-level bureaucrats may have led to stakeholders’ opposition and policy failure eventually:

“*The Government was the sole actor in the implementation of this policy, while the Ministry of Health was against it. No other organization was taken into consideration for designing as well as putting this policy into practice. The top-down approach may have hastened the policy implementation, but it will not last for long time*.” (National policy maker).

One of the main challenges to the implementation of policy was lack of full expansion of insurance coverage nationwide, prior to putting hospital autonomy into practice:

“We *focused on economic aspect of hospitals first, prior to attempting to expand insurance coverage universally. We should have done exactly the opposite: insuring people before creating autonomous hospitals”* (Former senior health policy maker).

“*In the years following this plan, hospital autonomy outpaced universal health insurance and caused serious problems*.” (Former minister of health).

#### The blanket approach (‘one size fits all’ approach)

The fee-for-service payment policy, which was endorsed by the MSI Act and was entirely implemented in all public hospitals across the country, aimed to increase the efficiency of autonomous hospitals. The majority of interviewees described this policy as the “blanket approach”, meaning that regardless of regional differences in terms of patient distribution and human resources, the MSI Act covered all public hospitals across the country. No well-developed plan existed about the distribution of diseases across the country and in different hospitals. Lack of thorough investigation and feasibility analysis before and during the implementation as well as treating all hospitals equally without categorizing them, led many hospitals face serious problems. This resulted in many disadvantaged regional hospitals to face many unwanted problems.

“*If such a hospital was managed based on the fee for service policy, how could it be compared to a hospital in central areas of the province*?” (Former senior national policy maker).

Our findings showed that Fee-for-service payment increased induced demand and moved hospitals towards creating hospital wards that are more profitable and with greater cost benefit:

“*Fee-for-service payment in hospitals increased some inappropriate behaviors of health providers as induced demand and receiving under table payments*” (Senior Policy maker).

Various bed occupancy rates, and special services provided by various hospitals, were two factors that could affect hospital’s revenue:

“*It is really meaningless for this policy to cover mental hospitals, burn centers, and other chronic diseases’ hospitals. Bed turnover is very low in such hospitals. Consequently, this approach would lead to low income and finally insolvency in such hospitals*.” (Former hospital manager).

To compensate insurance organizations’ failure in proper and timely allocation of resources among hospitals, it was decided to increase the authority of medical universities for collecting revenues from the insurance organizations, as well as allocating funds and revenues towards hospitals. However, medical universities did not have the right criteria for resource allocation, which gradually led to creation of a bargaining atmosphere between hospitals and medical universities to absorb more funds. Some interviewees thought that this led to unfair budget allocations, where some hospitals with the best performance were given the lowest priority. This in turn encouraged poor performance among hospitals:

“*I argue that the university allocated the best resources to the worst hospitals. In doing so, it discouraged better performing hospitals*.” (Former hospital manager).

#### Timing of implementation

Particular characteristics of the health system and hasty implementation led to the failure of hospital autonomy policy in Iran:

“*Hasty and unprofessional implementation of MSI Act pushed many hospitals toward bankruptcy and closure*.” (Former senior national official).

“*Our argument is that if we want to implement Medical Services Insurance Act, we should not suddenly impose the costs of the hospitals on the medical sciences universities, stopping the budget for running costs and telling them to cover via your revenues because the insurance is being implemented*” [September 1991, Parliament’s 3’rd period, Session400, Gazette No:14788]*.*

Let alone that much longer time was required for appropriate implementation of a policy with such nature:

“*The fact that we immediately implemented the hospital autonomy policy after the approval of MSI Act and anticipated a five-year period for complete implementation of the latter was so naïve*.” (Former advisor to the minister of health).

## Discussion

The Medical Services Insurance (MSI) Act was approved in 1994 and led to the establishment of Medical Services Insurance Organizations (MSIO) in the same year in Iran. The policy was an effort to implement hospitals’ autonomy and develop contracts, on the basis of fee-for-service arrangements between the MSIO and participating (public teaching) hospitals. The newly funded insurance organization (MSIO) was not an independent entity, and performed under the authority of MOHME until 2004, when it moved under the newly established ministry of Welfare and Social Security (currently: Ministry of Cooperatives, Labor and Social Affair). The underlying aim of MSI Act was splitting purchaser (insurance agents) from provider (MOHME) within the health system. We explored the reasons for hasty, incomplete, and unsuccessful implementation of MSI Act and its unexpected and sometimes adverse effects on the Iranian health system.

We found serious limitations in the design (policy content) and the conceptual framework of the policy, important contextual issues (implementation context), as well as its subsequent delivery and implementation (implementation process). The hospital autonomy policy was in essence a hospital financing policy. The policy did not fully acknowledge the contextual issues that distorted the effects of the financing recommendations, namely policy contents, which were incorporated into the policy. The fee-for-service payments and the medical tariffs structure increased hospital costs. The organizational limitations of the insurance organizations, hospitals and the medical universities resulted in their inabilities to monitor providers’ behavior and limit the costs. In fact, the hospitals and medical universities were incentivized to maintain physicians’ revenue-maximizing behaviors. The policy proponents also underestimated the strong oppositions of several stakeholder groups, and failed to incorporate any pilot or feasibility assessments to identify potential shortcomings. Such important shortcomings were augmented with a definitive mandate that made it impossible to revert a policy that was enshrined in the annual budget acts; and a hasty, top-down and ‘one-size-fits-all’ implementation approach. As a result, the policy makers did not find any appropriate opportunity to correct the shortcomings. Therefore, the policy implementation process was managed through reactive decisions to resolve identified problems.

### Coincidence of policies: the policy window

The implementation of hospital autonomy illustrates the importance of policies’ coincidence in pushing a complex policy into a health system. The establishment of MSIO, whose mandate was fulfilling universal coverage, accelerated the process of policy ratification and made the implementation of PPS more feasible. PPS aimed to increase efficiency and improve accountability in a publicly financed health system of Iran. Fundamental challenges hampered the policy implementation and impeded the achievement of its aims. Although hospitals received money for staff’s salary and overhead expenses from insurance organization, organizational deficits of health insurance system decreased hope and erode reliance on insurance organizations eventually.

In principle, both the price and the volume of services in fee for service contracts, must be prospectively determined by the purchasers [21]. Our findings highlighted that low experience of newly established insurance organizations; delayed payments; inappropriate and unrealistic medical tariffs [42] (existing conflict of interest in determining tariffs, lack of a sound accounting system in determining tariffs, and tariffs not being based on day to day costs and revenues), plus the weak capacity of hospitals, were serious challenges to the implementation of PPS policy and hospitals’ performance in Iran. Such shortages led to irrational relative value of health services, which in turn resulted in increasing OOP payment, as well as the spreading under table payments in order to run hospitals, both of which were seriously unwanted by policy makers.

### Policy makers’ view points and complexity of the health system

According to system thinking principle, “everything is connected to everything else”. [43] Implementing a reform or changing a policy is extremely complex; hence predicting the behavior of the health care system becomes difficult [4, 44].

Holistic approach to health system would enable policy makers to identify the high leverage points in the policy cycle to avoid policy resistance [45]. In formulating and implementing a policy, a systematic perspective helps policy makers to make decisions that are consistent with their long-term best interests and the long-term best interests of the system as a whole [43]. The hospital autonomy policy fulfilled only one dimension of PPS, while neglecting other dimensions. The readiness of purchasers and reducing information asymmetries between providers (hospitals) and purchasers (insurance bodies) are important prerequisites of a successful PPS policy [46]. Hospital autonomy policy expected the hospitals to act as economic units, while the policy ignored the need for the development of effective market regulations mechanisms.

In addition, the potential revenues of hospitals from the insurers did not match the financial needs and expectations of the teaching hospitals, as well as hospitals located in small towns and cities. The policy did not acknowledge different requirements of such hospitals [47]. As a result, hospitals - and subsequently the ministry of health and medical education - started lobbying the government and parliament to allocate other ‘public’ funds towards hospitals. Such practice was against the primary intention of the policy makers. The hospitals had no choice than funding themselves through other directions and moved towards financing through out of pocket payments by patients.

Hasty implementation of PPS policy and the limited time period (less than a year) that was spent to develop the purchasing processes, transforming the budget system, and preparing the cultural change requirements for both purchasers and providers, had some severe adverse consequences for the policy implementation [48]. Expecting the full implementation of the universal health insurance in five years time was far from reality. The MSIO failed to meet this target by 1999, as it still remains unmet [49]. Implementing universal health insurance is a gradual approach and requires meticulous planning [50–53].

### Policy triangle framework and hospital autonomy in Iran

Based on traditional stage heuristic framework for policy analysis, for any policy initiative to be effective, all four components (context, content, players and process) of policy must be taken into consideration [54, 55]. Understanding contextual opportunities, their complexity and constraints to policy change such as situational, structural, cultural and international or exogenous, which are specific for setting and time, help policy entrepreneurs and innovators move toward policy objectives [56, 57]. Our findings illustrated that none of these components were appropriately prepared for the implementation of fee for service policy in teaching hospitals in Iran. The creation of MSIO was an attempt to curb some of the main health system problems, i.e., improving efficiency, responding to patients’ expectations, making money following the patients, and improving access to medical care and public hospitals’ accountability. Our findings suggest that none of these goals were achieved as a result of the hospital autonomy policy in Iran. Implementing MSIO through PPS policy not only did not led to development of universal health coverage in Iran, but it actually brought people’s hope down for receiving high quality healthcare in public hospitals.

Policy development is a process, which happens over time and requires both intra- and inter-organizational coordination [58]. The role of actors (players), their underlying interests and the level of their commitments toward the desired policy or specific issue, and also understanding their perceptions and values is another important factor in developing and implementing a policy [11, 59, 60]. Government, as one of influential actors, did not take its full role in implementing hospital autonomy policy in Iran. The government took an important role in developing the policy, but it did not fully materialize its duty to fund the implementation of policy and support the insurance organizations.

Evidence shows managerial problems in hospitals and insurance organizations somewhat weigh in a balance with the positive effects and outcomes accruing to them [61]. Our findings implied the lack of training provided to both the new breed of hospital managers and insurers to undertake radically different roles within the Iranian healthcare system as another challenge in fully and successful development of the PPS policy. Obviously, it is unrealistic to expect health professionals to become expert managers over a night, and without adequate training about the new context and requirements of their new role.

### Limitations of this study

The retrospective nature of this policy study caused difficulties for interviewees in recalling the policy events, the trends and their causes and effects. The main documents of the study belonged to about two to three decades ago. There was no existing and comprehensive collection of relevant policy documents. We identified the collected documents via searching relevant libraries and following the leads that we found in both the documents and the interviews. Despite the efforts, we cannot claim that all the relevant documents have been covered in this study.

## Conclusions

In this paper, we reported a retrospective analysis to understand strengths and weaknesses of development and implementation of teaching hospitals’ autonomy in Iran. The MSI Act and establishment of MSIO, which aimed at hospitals’ autonomy and purchaser-provider split, were appropriately formulated policies. However, the implementation of those policies was problematic and ended up in some adverse consequences such as higher out of pocket expenditure. The policy led hospitals to behave as a profit-maximizing firm. Our evidence leads us to recommend gradual, cautious, and well-planned shift to a contract-based relationship within a hospital autonomy policy implementation, if at all. Any attempts to increase hospital’s revenue through dropping fee for service arrangements may lead to unwanted consequences for quality of health services, education purposes (in teaching hospitals) and eroding public trust in the health care system. Hence, careful consideration and appropriate preparation are required prior to implementing such reforms in any hospital setting. Monitoring the implementation process, identifying the challenges, and addressing shortcoming at the right time, are crucial prerequisites of successful implementation of hospital autonomy.

### Availability of data and materials

The datasets supporting the conclusions of this article are included within the article.

### Consent to participate and consent to publish

Oral consent to participate, record and publish the data collected from the participants was obtained at each interview by the interviewer. As also described in the Methods section, it was culturally inappropriate to request written consent given the participants’ high official governmental ranks.

## Appendix

### Interview guide

**Date and venue:**

**Name and position of interviewee**

1. Please explain your general perception of the hospital board of trustees’ policy (approved 2004) in Iran.

2. What were the aims and objectives of the policy? Why this policy was formulated and implemented?

3. How the policy entered to the policy agenda? By whom (individuals, groups or organizations)? How the policy was formulated and implemented? Which groups or organizations were opponents or proponents of the policy?

4. What is your opinion about implementation of the hospital board of trustees’ policy in the following of hospital autonomy policy?

5. Which financing policies were adopted in accordance with the policy?

6. To what extent do you think the policy was properly implemented and the aims were achieved?

7. Please, explain the strengths and weaknesses of the policy. Which aspects of the policy need to be changed or improved?

8. To what extent do you think the policy was clear (in the content, objectives, expected results and outcomes)?

9. Regarding the policy, to what extent do you think Iranian public hospitals and health system have had the capacity for proper implementation of the policy? Which essentials should have been considered before the implementation of the policy?

10. In what extent do you think implementation of the policy was based on evidence (local or global)?

11. What were the effects of the policy (positive and negative) on hospitals, health system and community?

12. From your point of view, why teaching hospitals were selected for the implementation of the policy.

13. Considering the fact that several years have passed since the implementation of the policy, what are the main problems of policy implementation in hospitals?

14. What are the effects of the policy on financing of teaching hospitals? What were the main reasons of changing financing method of the hospitals?

15. So far, have you received any feedback from policy-makers and stakeholders such as insurance organizations, medical council organization, medical tariff office and people regarding the implementation of the policy? Please discuss. How did you deal with these feedbacks?

16. Do you have any evidence to show the extent by which the policy has been accepted by the stakeholders?

17. Would you please, give your opinion about, whether the policy was successful or not? If your answer is no, explain why it has not been ended?

18. As a final question, is there any further issue you would like to add?

Note: The questions were adjusted based on each interviewee’s position in health system.

With special thanks for your invaluable contribution.

**Focus Group Discussion Guide**

1. To what extent do you think the hospital board of trustees’ policy was properly implemented in hospitals and the aims were achieved? Do you have any evidence? Please explain more.

2. To what extent do you think the policy was based on knowledge of “what works”? Please explain.

3. In your opinion, to what extent do you think the financing mechanism of the policy has been interpreted comprehensively and objectives, functions and consequences were clear?

4. To what extent do you think our health system have had capacity for implementing the policy? Before implementation of the policy what actions and activities should be implemented in hospitals?

5. Do you think implementation of the policy was based on evidence (local and global)? Please explain.

6. Please explain regarding positive and negative effects of the policy in teaching hospitals?

7. To what extent do you think proper and complete implementation of the policy will be effective in dealing with the current challenges of the hospitals and the health system?

## Footnotes

1 http://www.maxqda.com/products/maxqda10
